# Gastrin-Releasing Peptide Receptor Antagonist [^68^Ga]RM2 PET/CT for Staging of Pre-Treated, Metastasized Breast Cancer

**DOI:** 10.3390/cancers13236106

**Published:** 2021-12-03

**Authors:** Kerstin Michalski, Lars Kemna, Jasmin Asberger, Anca L. Grosu, Philipp T. Meyer, Juri Ruf, Tanja Sprave

**Affiliations:** 1 Department of Nuclear Medicine, Medical Center, Faculty of Medicine, University of Freiburg, Hugstetter Straße 55, 79106 Freiburg, Germany; lars.kemna@uniklinik-freiburg.de (L.K.); philipp.meyer@uniklinik-freiburg.de (P.T.M.); juri.ruf@uniklinik-freiburg.de (J.R.); 2 Department of Obstetrics and Gynecology, Medical Center, Faculty of Medicine, University of Freiburg, Hugstetter Straße 55, 79106 Freiburg, Germany; jasmin.asberger@uniklinik-freiburg.de; 3 Department of Radiation Oncology, Medical Center, Faculty of Medicine, University of Freiburg, Hugstetter Straße 55, 79106 Freiburg, Germany; anca.grosu@uniklinik-freiburg.de (A.L.G.); tanja.sprave@uniklinik-freiburg.de (T.S.)

**Keywords:** RM2 PET/CT, metastasized breast cancer, theranostics, oligometastatic, ablative radiation therapy

## Abstract

**Simple Summary:**

[^68^Ga]RM2 positron emission tomography (PET)/computed tomography (CT) has shown to be a promising imaging method for primary breast cancer (BC) with positive estrogen receptor (ER) status, but it has not been tested for visualization of metastasized recurrent or progressive BC. The aim of this pilot study was to assess tumor visualization using [^68^Ga]RM2 PET/CT in patients with pre-treated ER-positive BC and suspected metastases. Eight female patients with initial ER-positive, pre-treated BC were included in this retrospective study. Strong RM2 binding was found in all metastatic lesions of six patients, whereas two patients were rated RM2-negative. Our data suggest that RM2 binding is maintained in the majority of patients with advanced disease stage of pre-treated ER-positive BC. Thus, [^68^Ga]RM2 PET/CT could support treatment decision in these patients, radiotherapy planning in oligometastatic patients or selection of patients for RM2 radioligand therapy.

**Abstract:**

Background: Positron emission tomography (PET)/computed tomography (CT) using the gastrin-releasing peptide receptor antagonist [^68^Ga]RM2 has shown to be a promising imaging method for primary breast cancer (BC) with positive estrogen receptor (ER) status. This study assessed tumor visualization by [^68^Ga]RM2 PET/CT in patients with pre-treated ER-positive BC and suspected metastases. Methods: This retrospective pilot study included eight female patients with initial ER-positive, pre-treated BC who underwent [^68^Ga]RM2 PET/CT. Most of these patients (seven out of eight; 88%) were still being treated with or had received endocrine therapy. [^68^Ga]RM2 PET/CTs were visually analyzed by two nuclear medicine specialists in consensus. Tumor manifestations were rated qualitatively (i.e., RM2-positive or RM2-negative) and quantitatively using the maximum standardized uptake value (SUVmax). SUVmax values were compared between the two subgroups (RM2-positive vs. RM2-negative). Results: Strong RM2 binding was found in all metastatic lesions of six patients (75%), whereas tracer uptake in all metastases of two patients (25%) was rated negative. Mean SUVmax of RM2-positive metastases with the highest SUVmax per patient (in lymph node and bone metastases; 15.8 ± 15.1 range: 3.7–47.8) was higher than mean SUVmax of the RM2-negative metastases with the highest SUVmax per patient (in bone metastases; 1.6 ± 0.1, range 1.5–1.7). Conclusions: Our data suggest that RM2 binding is maintained in the majority of patients with advanced disease stage of pre-treated ER-positive BC. Thus, [^68^Ga]RM2 PET/CT could support treatment decision in these patients, radiotherapy planning in oligometastatic patients or selection of patients for RM2 radioligand therapy. Further studies with larger patient cohorts are warranted to confirm these findings.

## 1. Introduction

Breast cancer (BC) still represents the most common type of cancer in women worldwide [1]. In case of distant metastases, systemic therapies, such as endocrine therapy, chemotherapy, targeted drugs and immunotherapy, are the main treatments [2]. However, recent data have shown the benefit of side-directed therapy in case of low metastatic burden for cancer control and survival outcomes in other tumor entities [3,4,5,6,7]. Oligometastatic disease is considered an intermediate stage between localized and systemic metastatic disease [8]. It typically includes a maximum of five metastases [9] and is diagnosed by imaging alone [10]. The detection of an oligometastatic stage of disease is crucial before ablative radiotherapy, especially if associated with a potentially curative intent. Conventional imaging lacks information about vital tumor cells, which is especially challenging in case of sclerotic bone lesions. In analogy to highly accurate molecular imaging techniques in other tumor entities (e.g., prostate cancer) [6,11], [^68^Ga]RM2 positron emission tomography (PET)/computed tomography (CT) targeting the gastrin-releasing peptide receptor (GRPR) could help detecting the oligometastatic stage in patients with BC and might, thus, improve accurate stratification of this patient group.

The GRPR is a G-protein-coupled receptor and is part of the bombesin receptor family [12,13]. Its physiological ligand gastrin-releasing peptide as well as the receptor appear to influence tumor growth and carcinogenesis [14,15,16]. GRPR is overexpressed in various human tumor entities such as BC or prostate cancer and represents a promising target for PET imaging and radionuclide therapy [17,18,19,20]. Using immunohistochemistry in a large series of primary BC samples, Dalm et al. have shown an association between estrogen receptor (ER) and GRPR at the messenger ribonucleic acid level [21]. GRPR overexpression was observed in 83.2% of ER-positive tumors, but only in 12% of ER-negative tumors [22]. [^68^Ga]RM2 has shown to have a significantly higher tracer uptake in ER-positive tumors compared to [^18^F]Fluoro-2-deoxy-D-glucose ([^18^F]FDG) in another ex vivo study, in particular in tumors with low proliferation [23]. PET/CT using the GRPR antagonist [^68^Ga]RM2 has shown to be a promising imaging ligand for staging of primary ER-positive BC. In recent studies of our group, we demonstrated that [^68^Ga]RM2 visualized 13 of 14 ER-positive breast tumors in untreated patients showing a high correlation between [^68^Ga]RM2 binding and ER expression [20]. Furthermore, we found that residual uptake of [^68^Ga]RM2 in ER-positive primary BC correlated well with residual vital tumor size after neoadjuvant chemotherapy [24].

Some antiestrogens (such as Fulvestrant) affect ER stability and cause a downregulation of the receptor protein [25]. It is unclear whether antiestrogens also affect GRPR expression and, thus, tumor visualization by [^68^Ga]RM2. Ellis et al. found a low fraction of about 10% of tumors that switch to an ER-negative status after endocrine therapy [26]. Previous studies on GRPR ligands included only patients with untreated primary BC [20,27] or visualization of treatment response to neoadjuvant chemotherapy [24].

To our knowledge, [^68^Ga]RM2 PET/CT has not been tested for visualization of metastasized recurrent or progressive BC. Especially, the effects of endocrine therapy on tumor binding of [^68^Ga]RM2 are unknown. The aim of this pilot study was to assess tumor visualization using [^68^Ga]RM2 PET/CT in patients with pre-treated ER-positive BC and suspected metastases.

## 2. Materials and Methods

Patients with pre-treated initially ER-positive BC who underwent [^68^Ga]RM2 PET/CT between October 2019 and November 2020 were screened for eligibility. All patients had oligometastatic or oligoprogressive bone metastases on conventional imaging and were examined with [^68^Ga]RM2 PET/CT on compassionate use basis before planned radiotherapy in order to rule out further potential occult vital metastases. Inclusion criteria were recurrent or progressive metastatic BC with initially positive ER receptor status (ER ≥ 40%). At least one metastasis per patient had to be confirmed by either histopathology or by distinct appearance with size progression on CT (lymph node and visceral metastases) or (if available) by distinct appearance on current magnetic resonance imaging (MRI) or [^18^F]FDG PET (bone metastases). All [^68^Ga]RM2 PET scans had to be performed on the same PET scanner. All patients gave written informed consent. The institutional ethical review board (20-1050) approved the data analysis.

Tracer synthesis was performed as already described previously [20]. Injected activity of [^68^Ga]RM2 was on average 201 ± 8 (192–211) MBq. Whole-body PET/CT scans were performed 1 h after injection of [^68^Ga]RM2 with a Vereos Digital PET/CT (Philips Healthcare, Cleveland, OH, USA) with either a full-dose contrast-enhanced diagnostic CT or a low-dose CT (depending on previous imaging). PET/CT images included the trunk from skull base to mid-thigh with a 2 min scan time per bed position. All images were reconstructed with a vendor-specific time-of-flight iterative reconstruction algorithm (BLOB-OS-TF [28]) with three iterations and nine subsets (relaxation parameter 0.35) and a voxel size of 2 × 2 × 2 mm^3^. The spatial resolution of the reconstructed Gallium-68 PET image is about 5 mm full width half maximum (FWHM).

Two nuclear medicine specialists (J.R. and L.K.), who had more than 15 years of experience, visually analyzed all PET/CT scans using IMPAX EE (Agfa Health Care, Bonn, Germany). Lesions were rated suspicious for metastases and RM2-positive or -negative in consensus. Metastases were rated in conjunction of the PET and CT scan. Other pre-existing imaging modalities, such as older CT scans or a current MRI or [^18^F]FDG PET scan (if available), were also included in the visual assessment. Positivity was defined as non-physiological high tracer uptake relative to local background. Another nuclear medicine specialist (KM) with 5 years of experience performed a volume-of-interest (VOI) analysis of the [^68^Ga]RM2 PET scans using PMOD (PMOD Technologies, Zurich, Switzerland). The lesions rated suspicious for malignancy (both RM2-positive or -negative) were delineated under CT guidance on all transaxial slices showing the lesions and the maximum standardized uptake value (SUVmax) was determined. Statistical analyses were performed using SPSS software ver. 27.0 (IBM, Armonk, NY, USA). Descriptive data are presented as mean ± standard deviation and range. For descriptive comparison between subgroups (RM2-positive vs. RM2-negative), the metastasis with the highest SUVmax per patient was used.

## 3. Results

### 3.1. Patient Characteristics

Eight of ten patients screened met the inclusion criteria. Two patients were excluded as they presented with older sclerotic bone lesions which could not be confirmed as vital metastases by either tumor growth on current CT or by a distinct appearance on other imaging modalities. The characteristics of all eight included patients are given in Table 1. Mean patient age was 69 years (36–82 years). Mean time since diagnosis of BC was 12.5 years (0.5–32 years). Most of the patients (seven out of eight; 88%) were still treated with (*n* = 5) or had received endocrine therapy (*n* = 2).

### 3.2. Image Analysis

**Figure 1:**
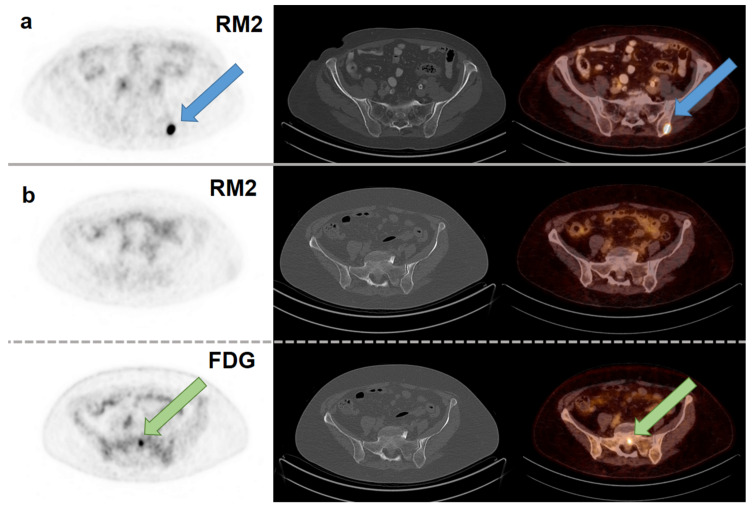
Axial slices of PET scans (first column), CT scans (second column) and fusion images (third column). (**a**) [^68^Ga]RM2 PET/CT of a 73-year-old patient (patient no. 3) with a bone metastasis in the left iliac bone with intense RM2 binding and not seen on CT (blue arrow; SUVmax 32.1; first row). (**b**) [^68^Ga]RM2 PET/CT of a 75-year-old patient (patient no. 5) with a bone metastasis in the sacrum without RM2 binding and not seen on CT (SUVmax 1.5; second row). [^18^F]FDG PET/CT of the same patient with intense hypermetabolism in the sacrum (green arrow; SUVmax 5.8; third row). Note the physiologically higher tracer uptake in healthy bone on [^18^F]FDG PET. Fixed inversed grey-scale images are displayed with an SUV window of 0–5.

Strong RM2 binding was found in all metastases in six out of eight patients (75%). These patients presented with lymph node, bone, and liver metastases (Table 2). Negative [^68^Ga]RM2 PET scans were found in two patients (25%) with either lymph node (short axis ≥ 1 cm on CT) or bone metastases (verified by MRI and [^18^F]FDG PET). One of these patients with RM2-negative metastases (patient no. 2) in lymph nodes and bones presented with a new pleural effusion 4 months after [^68^Ga]RM2 PET/CT, which contained ER-negative BC cells. Figure 1 shows examples of a positive and a negative [^68^Ga]RM2 PET/CT. Quantification of tracer uptake confirmed visual assessment of RM2-binding. Mean SUVmax of RM2-positive metastases with the highest SUVmax per patient (in lymph node and bone metastases; 15.8 ± 15.1 range: 3.7–47.8) was higher than mean SUVmax of the RM2-negative metastases with the highest SUVmax per patient (in bone metastases; 1.6 ± 0.1, range 1.5–1.7). Oligometastatic stage of disease (≤5 metastases) was ruled out in three out of six RM2-positive patients.

## 4. Discussion

Our results suggest a persistent high tumor binding of [^68^Ga]RM2 in patients with initial high ER status and who were currently treated or had been treated with different endocrine therapies. In line with this, the analysis of immunohistopathology showed maintained high ER expression in seven out of eight patients (88%). A loss of ER expression was found in one patient (patient no. 2), who was also rated RM2-negative, after [^68^Ga]RM2 PET/CT. A discordance of hormone receptor status in primary BC and metachronous distant metastases has been demonstrated in many studies, with changes occurring in the ER status in 10% to 30% cases [29]. In addition, tumor heterogeneity can be found in different (metachronous) metastases of the same patient, with discordant rates of the ER status ranging from 10.9% to 27.5% [30,31]. However, most studies about the discordant hormone receptor status in primary tumor and distant metastases are retrospective and include tissue samples from different time points. Little is known about the possible synchronous receptor heterogeneity in multiple distant metastases, which could lead to RM2-positive and -negative metastases within the same patient. Using 16α-[^18^F]fluoro-17β-estradiol (FES) PET compared to [^18^F]FDG PET, Boers et al. found a heterogeneous [^18^F]FES binding in 20 of 30 patients with metastasized BC [32], which indicates a possible synchronous ER heterogeneity in advanced BC patients. However, GRPR expression was maintained in the majority of patients (75%) in our study group and no discordant ratings (i.e., RM2-positive or -negative) occurred within the same patient. Hence, [^68^Ga]RM2 PET/CT appears to be a useful imaging modality in staging and re-staging of initial ER-positive BC.

Furthermore, GRPR is also a promising target for theranostic approaches, since RM2 can be labeled with beta-emitting radiometals for therapeutic purposes. Kurth et al. showed that radioligand therapy using [^177^Lu]RM2 was safe in men with metastasized castration-resistant prostate cancer and found high absorbed tumor doses with a maximum absorbed dose in bone, lymph node, visceral and hepatic metastases of 19.2 Gy, 12.1 Gy, 9.5 Gy and 1.5 Gy, respectively [33]. Currently, no data exist for the use of [^177^Lu]RM2 in patients with metastasized BC, but our data suggest that [^177^Lu]RM2 might also be an option for patients with pre-treated, metastasized BC.

[^68^Ga]RM2 PET/CT ruled out an oligometastatic stage of disease (≤5 metastases) in half of the RM2-positive patients (three out of six) and could, therefore, be used for patient stratification. Recent studies have already shown the safety and a benefit of ablative radiotherapy in oligometastatic patients [34,35,36]. Both the number of metastases and organs affected appear to have an impact on progression-free survival [37]. The growing interest in the treatment concept of oligometastatic disease has led to numerous studies, which will provide their results in the coming years [38,39]. Nevertheless, the detection of the oligometastatic stage remains the key challenge. In contrast to [^18^F]FDG PET, a superior distinction of malignancy and inflammation can be assumed for [^68^Ga]RM2 PET due to its specific receptor binding. Our pilot study demonstrated the feasibility of detecting oligometastatic stage and may improve proper stratification. Further confirmatory studies evaluating the role of RM2-based metastasis-directed therapy in this risk population are required.

This study suffers from some limitations. The first is the limited cohort size in this retrospective pilot study and prospective studies with larger patient cohorts are warranted. Second, the time interval between a current biopsy and PET/CT differed considerably, with two patients having their last biopsy more than one year before PET/CT. One of these patients was rated RM2-negative, which could be due to a loss of ER or GRPR expression of the metastases after the beginning of endocrine therapy. Furthermore, tumor heterogeneity in the different metastases as mentioned above is possible, although visual rating of intraindividual metastases showed a consistent uptake intensity. For comparative reasons, semiquantification of the metastases was limited to the hottest lesions per patients. Third, no histopathological proof of all metastases exists, as this would not have been ethically feasible.

## 5. Conclusions

Our data suggest that RM2 binding is maintained in the majority of patients with advanced disease stage of pre-treated, ER-positive BC. Thus, [^68^Ga]RM2 PET/CT could support treatment decision in these patients, radiotherapy planning in oligometastatic patients or selection of patients for RM2 radioligand therapy. Further investigations with larger patient cohorts are warranted to confirm these findings.

## Figures and Tables

**Table 1:** Patient characteristics.

Pat. No.	Type/Side	Previous Treatment (Duration: Years)	Current Treatment (Duration: Years)	Time Since Initial Diagnosis (Years)
1	NST/left	Sentinel-LND, NAC, BCS, adj. Rtx, Tamoxifen	Tamoxifen (5)	5
2	ILC/right	-	Palbociclib, Letrozole (2)	2
3	NST/right	BCS, Sentinel-LND, adj. Rtx, Anastrozole (5)	-	6
4	n.a./n.a.	BSC, adj. Rtx, adj. Ctx, Letrozole (10)	-	16
5	ILC/right	Mastectomy, surgery of local relapse (2×), Exemestan (14)	Tamoxifen (1.5)	32
6	NST/right	Mastectomy, LND, adj. Rtx	Anastrozole (0.5)	0.5
7	NST/left	BCS, adj. Rtx, adj. Ctx, Rtx different bone metastases, Everolimus, Exemestan (5) Fulvestrant (1.5), Palbociclib, Capecitabine	Tamoxifen (0.5)	27
8	NST/right	BCS, Sentinel-LND, adj. Rtx	-	12

Abbreviations: NST: invasive carcinoma of no special type, ILC: invasive lobular carcinoma, LND: lymph node dissection, NAC: neoadjuvant chemotherapy, BCS: breast-conserving surgery, adj.: adjuvant, Rtx: radiotherapy, Ctx: chemotherapy, n.a.: not available.

**Table 2:** Findings on [^68^Ga]RM2 PET/CT compared to current biopsy.

Pat. No.	Rating RM2 PET	Sites of Metastatic Disease	No. of Metastases	Current Biopsy		
				Site	ER Status	Time Interval to PET (Months)
1	Positive	OSS, LN, HEP	7	HEP	>90%	5 (after)
2	Negative	OSS, LN	>10	Pleural effusion	0%	4 (after)
3	Positive	OSS	5	OSS	>90%	1 (before)
4	Positive	OSS	1	OSS	>90%	1 (before)
5	Negative	OSS	4 *	Local relapse	>90%	17 (before)
6	Positive	OSS, LN	9	Primary tumor	>90%	4 (before)
7	Positive	OSS, LN, HEP	>30	OSS	>90%	22 (before)
8	Positive	OSS	1	OSS	>90%	1 (before)

Abbreviations: OSS: bone, HEP: liver, LN: lymph node; * verified by [^18^F]FDG PET/MRI.

## Data Availability

The data used in this analysis are available with the authors’ permission upon request and ethical approval.

## References

[1] Bray F., Ferlay J., Soerjomataram I., Siegel R.L., Torre L.A., Jemal A. (2018). Global cancer statistics 2018: Globocan estimates of incidence and mortality worldwide for 36 cancers in 185 countries. CA Cancer J. Clin..

[2] American Cancer Society Treatment of Breast Cancer by Stage. https://www.cancer.org/cancer/breast-cancer/treatment/treatment-of-breast-cancer-by-stage.html.

[3] Gomez D.R., Tang C., Zhang J., Blumenschein G.R., Hernandez M., Lee J.J., Ye R., Palma D.A., Louie A.V., Camidge D.R. (2019). Local consolidative therapy vs. maintenance therapy or observation for patients with oligometastatic non-small-cell lung cancer: Long-term results of a multi-institutional, phase II, randomized study. J. Clin. Oncol..

[4] Lehrer E.J., Singh R., Wang M., Chinchilli V.M., Trifiletti D.M., Ost P., Siva S., Meng M.B., Tchelebi L., Zaorsky N.G. (2021). Safety and survival rates associated with ablative stereotactic radiotherapy for patients with oligometastatic cancer: A systematic review and meta-analysis. JAMA Oncol..

[5] Ost P., Reynders D., Decaestecker K., Fonteyne V., Lumen N., De B.A., Lambert B., Delrue L., Bultijnck R., Claeys T. (2018). Surveillance or metastasis-directed therapy for oligometastatic prostate cancer recurrence: A prospective, randomized, multicenter phase ii trial. J. Clin. Oncol..

[6] Phillips R., Shi W.Y., Deek M., Radwan N., Lim S.J., Antonarakis E.S., Rowe S.P., Ross A.E., Gorin M.A., Deville C. (2020). Outcomes of observation vs stereotactic ablative radiation for oligometastatic prostate cancer: The oriole phase 2 randomized clinical trial. JAMA Oncol..

[7] Siva S., Bressel M., Murphy D.G., Shaw M., Chander S., Violet J., Tai K.H., Udovicich C., Lim A., Selbie L. (2018). Stereotactic Abative Body Radiotherapy (SABR) for oligometastatic prostate cancer: A prospective clinical trial. Eur. Urol..

[8] Hellman S., Weichselbaum R.R. (1995). Oligometastases. J. Clin. Oncol..

[9] Guckenberger M., Lievens Y., Bouma A.B., Collette L., Dekker A., deSouza N.M., Dingemans A.C., Fournier B., Hurkmans C., Lecouvet F.E. (2020). Characterisation and classification of oligometastatic disease: A European society for radiotherapy and oncology and European organisation for research and treatment of cancer consensus recommendation. Lancet Oncol..

[10] Lecouvet F.E., Oprea-Lager D.E., Liu Y., Ost P., Bidaut L., Collette L., Deroose C.M., Goffin K., Herrmann K., Hoekstra O.S. (2018). Use of modern imaging methods to facilitate trials of metastasis-directed therapy for oligometastatic disease in prostate cancer: A consensus recommendation from the EORTC imaging group. Lancet Oncol..

[11] Ong W.L., Koh T.L., Lim J.D., Chao M., Farrugia B., Lau E., Khoo V., Lawrentschuk N., Bolton D., Foroudi F. (2019). Prostate-specific membrane antigen-positron emission tomography/computed tomography (PSMA-PET/CT)-guided stereotactic ablative body radiotherapy for oligometastatic prostate cancer: A single-institution experience and review of the published literature. BJU Int..

[12] Hohla F., Schally A.V. (2010). Targeting gastrin releasing peptide receptors: New options for the therapy and diagnosis of cancer. Cell Cycle.

[13] Ramos-Álvarez I., Moreno P., Mantey S.A., Nakamura T., Nuche-Berenguer B., Moody T.W., Coy D.H., Jensen R.T. (2015). Insights into bombesin receptors and ligands: Highlighting recent advances. Peptides.

[14] Yano T., Pinski J., Groot K., Schally A.V. (1992). Stimulation by bombesin and inhibition by bombesin/gastrin-releasing peptide antagonist RC-3095 of growth of human breast cancer cell lines. Cancer Res..

[15] Nelson J., Donnelly M., Walker B., Gray J., Shaw C., Murphy R.F. (1991). Bombesin stimulates proliferation of human breast cancer cells in culture. Br. J. Cancer.

[16] Lango M.N., Dyer K.F., Lui V.W.Y., Gooding W.E., Gubish C., Siegfried J.M., Grandis J.R. (2002). Gastrin-releasing peptide receptor-mediated autocrine growth in squamous cell carcinoma of the head and neck. J. Natl. Cancer Inst..

[17] Reubi J.C., Wenger S., Schmuckli-Maurer J., Schaer J.-C., Gugger M. (2002). Bombesin receptor subtypes in human cancers: Detection with the universal radioligand (125)I-D-TYR(6), beta-ALA(11), PHE(13), NLE(14) bombesin(6-14). Clin. Cancer Res..

[18] Reubi J.C., Fleischmann A., Waser B., Rehmann R. (2011). Concomitant vascular GRP-receptor and VEGF-receptor expression in human tumors: Molecular basis for dual targeting of tumoral vasculature. Peptides.

[19] Wieser G., Mansi R., Grosu A.L., Schultze-Seemann W., Dumont-Walter R.A., Meyer P.T., Maecke H.R., Reubi J.C., Weber W.A. (2014). Positron emission tomography (PET) imaging of prostate cancer with a gastrin releasing peptide receptor antagonist--from mice to men. Theranostics.

[20] Stoykow C., Erbes T., Maecke H.R., Bulla S., Bartholomä M., Mayer S., Drendel V., Bronsert P., Werner M., Gitsch G. (2016). Gastrin-releasing Peptide receptor imaging in breast cancer using the receptor antagonist (68)Ga-RM2 and PET. Theranostics.

[21] Dalm S.U., Martens J.W.M., Sieuwerts A.M., van Deurzen C.H.M., Koelewijn S.J., de Blois E., Maina T., Nock B.A., Brunel L., Fehrentz J.-A. (2015). In vitro and in vivo application of radiolabeled gastrin-releasing peptide receptor ligands in breast cancer. J. Nucl. Med..

[22] Morgat C., MacGrogan G., Brouste V., Vélasco V., Sévenet N., Bonnefoi H., Fernandez P., Debled M., Hindié E. (2017). Expression of gastrin-releasing peptide receptor in breast cancer and its association with pathologic, biologic, and clinical parameters: A study of 1432 primary tumors. J. Nucl. Med..

[23] Morgat C., Schollhammer R., MacGrogan G., Barthe N., Vélasco V., Vimont D., Cazeau A.-L., Fernandez P., Hindié E. (2019). Comparison of the binding of the gastrin-releasing peptide receptor (GRP-R) antagonist 68Ga-RM2 and 18F-FDG in breast cancer samples. PLoS ONE.

[24] Michalski K., Stoykow C., Bronsert P., Juhasz-Böss I., Meyer P.T., Ruf J., Erbes T., Asberger J. (2020). Association between gastrin-releasing peptide receptor expression as assessed with [68Ga]Ga-RM2 PET/CT and histopathological tumor regression after neoadjuvant chemotherapy in primary breast cancer. Nucl. Med. Biol..

[25] Clarke R., Tyson J.J., Dixon J.M. (2015). Endocrine resistance in breast cancer--An overview and update. Mol. Cell. Endocrinol..

[26] Ellis M.J., Tao Y., Luo J., A’Hern R., Evans D.B., Bhatnagar A.S., Chaudri R.H.A., von K.A., Miller W.R., Smith I. (2008). Outcome prediction for estrogen receptor-positive breast cancer based on postneoadjuvant endocrine therapy tumor characteristics. J. Natl. Cancer Inst..

[27] Zang J., Mao F., Wang H., Zhang J., Liu Q., Peng L., Li F., Lang L., Chen X., Zhu Z. (2018). 68Ga-NOTA-RM26 PET/CT in the evaluation of breast cancer: A pilot prospective study. Clin. Nucl. Med..

[28] Wang W., Hu Z., Gualtieri E.E., Parma M.J., Walsh E.S., Sebok D., Hsieh Y.-L., Tung C.-H., Song X., Griesmer J.J., Phlips B. (2006). Systematic and distributed time-of-flight list mode pet reconstruction. IEEE Nuclear Science Symposium Conference Record, Proceedings of the Nuclear Science Symposium, Medical Imaging Conference, 15th International Workshop on Room Temperature Semiconductor X- and Gamma-Ray Detectors, Special Focus Workshops, Town and Country Resort & Convention Center, San Diego, CA, USA, 29 October–4 November 2006.

[29] Rossi S., Basso M., Strippoli A., Dadduzio V., Cerchiaro E., Barile R., D'Argento E., Cassano A., Schinzari G., Barone C. (2015). Hormone receptor status and HER2 expression in primary breast cancer compared with synchronous axillary metastases or recurrent metastatic disease. Clin. Breast Cancer.

[30] Hoefnagel L.D.C., van der Groep P., van de Vijver M.J., Boers J.E., Wesseling P., Wesseling J., van der Wall E., van Diest P.J. (2013). Discordance in ERα, PR and HER2 receptor status across different distant breast cancer metastases within the same patient. Ann. Oncol..

[31] Chen R., Qarmali M., Siegal G.P., Wei S. (2020). Receptor conversion in metastatic breast cancer: Analysis of 390 cases from a single institution. Mod. Pathol..

[32] Boers J., Venema C.M., de Vries E.F.J., Glaudemans A.W.J.M., Kwee T.C., Schuuring E., Martens J.W.M., Sjoerd G.E., Hospers G.A.P., Schröder C.P. (2020). Molecular imaging to identify patients with metastatic breast cancer who benefit from endocrine treatment combined with cyclin-dependent kinase inhibition. Eur. J. Cancer.

[33] Kurth J., Krause B.J., Schwarzenböck S.M., Bergner C., Hakenberg O.W., Heuschkel M. (2019). First-in-human dosimetry of gastrin-releasing peptide receptor antagonist 177LuLu-RM2: A radiopharmaceutical for the treatment of metastatic castration-resistant prostate cancer. Eur. J. Nucl. Med. Mol. Imaging.

[34] Trovo M., Furlan C., Polesel J., Fiorica F., Arcangeli S., Giaj-Levra N., Alongi F., Del Conte A., Militello L., Muraro E. (2018). Radical radiation therapy for oligometastatic breast cancer: Results of a prospective phase II trial. Radiother. Oncol..

[35] David S., Tan J., Savas P., Bressel M., Kelly D., Foroudi F., Loi S., Siva S. (2020). Stereotactic ablative body radiotherapy (SABR) for bone only oligometastatic breast cancer: A prospective clinical trial. Breast.

[36] Steenbruggen T.G., Schaapveld M., Horlings H.M., Sanders J., Hogewoning S.J., Lips E.H., Vrancken P.M.T., Kok N.F., Wiersma T., Esserman L. (2021). Characterization of oligometastatic disease in a real-world nationwide cohort of 3447 patients with de novo metastatic breast cancer. JNCI Cancer Spectr..

[37] Milano M.T., Katz A.W., Zhang H., Huggins C.F., Aujla K.S., Okunieff P. (2019). Oligometastatic breast cancer treated with hypofractionated stereotactic radiotherapy: Some patients survive longer than a decade. Radiother. Oncol. J. Eur. Soc. Ther. Radiol. Oncol..

[38] Krug D., Vonthein R., Illen A., Olbrich D., Barkhausen J., Richter J., Klapper W., Schmalz C., Rody A., Maass N. (2021). Metastases-directed radiotherapy in addition to standard systemic therapy in patients with oligometastatic breast cancer: Study protocol for a randomized controlled multi-national and multi-center clinical trial (OLIGOMA). Clin. Transl. Radiat. Oncol..

[39] Alomran R., White M., Bruce M., Bressel M., Roache S., Karroum L., Hanna G.G., Siva S., Goel S., David S. (2021). Stereotactic radiotherapy for oligoprogressive ER-positive breast cancer (AVATAR). BMC Cancer.

